# The effect of home visits as an additional recruitment step on the composition of the final sample: a cross-sectional analysis in two study centers of the German National Cohort (NAKO)

**DOI:** 10.1186/s12874-021-01357-z

**Published:** 2021-08-23

**Authors:** Lilian Krist, Ahmed Bedir, Julia Fricke, Alexander Kluttig, Rafael Mikolajczyk

**Affiliations:** 1 Institute of Social Medicine, Epidemiology and Health Economics, Charité-Universitätsmedizin, Berlin, Germany; 2 Department of Radiation Oncology, Health Services Research Group, University Hospital Halle (Saale), Halle (Saale), Germany; 3 Institute of Medical Epidemiology, Biometry, and Informatics, Martin Luther University Halle-Wittenberg, Halle (Saale), Germany

**Keywords:** Response rate, Response proportion, Non-response bias, Mixed-mode design, Recruitment strategy, Home visits, Turkish, migrants

## Abstract

**Background:**

Participation in epidemiologic studies has been declining over the last decades. In addition to postal invitations and phone calls, home visits can be conducted to increase participation. The aim of this study was therefore to evaluate the effects of home visits in terms of response increase and composition of the additionally recruited and final sample.

**Methods:**

In the framework of the German National Cohort (NAKO) recruitment process, two of 18 study centers, Halle (Saale) and Berlin-Center, performed home visits as additional recruitment step after postal invitation and reminders. Response increase was calculated and differences between participants recruited via home visits and standard recruitment were examined. Proportions are presented as percentages with 95%-confidence intervals.

**Results:**

In the general population in Halle, 21.3-22.8% participated after postal invitation and two reminders in the five assessed recruitment waves. The increase of the overall response was 2.8 percentage points (95%confidence interval: 1.9-4.0) for home visits compared to 2.4 percentage points (95%CI: 1.7-3.3) for alternatively sent third postal reminder. Participants recruited via home visits had similar characteristics to those recruited via standard recruitment. Among persons of Turkish descent in Berlin-Center site of the NAKO, home visits conducted by native speakers increased the participation of women, persons living together with their partner, were born in Turkey, had lower German language skills, lower-income, lower education, were more often smokers and reported more often diabetes and depression to a degree which changed overall estimates for this subsample.

**Conclusions:**

As an additional recruitment measure in the general population, home visits increased response only marginally, and the through home visits recruited participants did not differ from those already recruited. Among persons with migration background, home visits by a native speaker increased participation of persons not reached by the standard recruitment, but the effects of using a native speaker approach could not be separated from the effect of home visits.

**Supplementary Information:**

The online version contains supplementary material available at 10.1186/s12874-021-01357-z.

## Background

Participation in studies has been steadily declining over the past few decades [1–3]. While the Framingham Heart Study (first wave 1948-1950) as one of the first epidemiological cohort studies reported a response proportion of 68.6% [4, 5], a much lower response is reported for more recent large cohort studies, e.g. “UK Biobank”, started in 2006 in the UK, or “NAKO”, started in 2014 in Germany [6–10].

Various reasons are likely to contribute to this decline (e.g. lower social engagement in general, higher workload, more relocations) [1, 11]. In any study type, a low response can result in nonresponse bias, a form of selection bias, caused by a non-random participation, with differences between those who participate and those who do not [12, 13]. When the response is high, a small group of non-participants is unlikely to change the overall estimates. When the overall response decreases, it still can be useful to increase participation of less represented groups of the population to be able to calculate weighted estimates. Since it is unlikely that we will be able to reach the participation level of the older studies in the future, the practical question is about the incremental value of additional efforts to increase response. This can vary among populations studied, for example, the mechanisms of response can be different in the general population, patient samples, or among migrant groups due to the additional difficulty of language barriers [2, 14, 15]. Different strategies to increase participation were proposed by several authors, including additional reminders or using various ways of approaching participants, for example, phone calls or home visits in addition to invitations via regular mail [16–18].

The aim of this study was therefore to investigate the effect of home visits as an additional step in the recruitment process a) on the response proportion and b) on the characteristics of the study population among a general population and migrants.

## Methods

### Study design and participants

We used data from two study centers of the German National Cohort (NAKO). The study design of the NAKO has been described in detail elsewhere [7, 19, 20]. Briefly, NAKO is a population-based prospective study that included more than 205.000 participants between 20 and 69 years of age at baseline from 18 study centers all over Germany. The overarching aim of the NAKO is to identify risk factors for common diseases such as cardiovascular diseases, diabetes, cancer, and neuropsychiatric, infectious, and musculoskeletal diseases including socio-economic, lifestyle-related, psychosocial, occupational, and environmental factors. The study was approved by the ethical review committees of all participating study centers including the Charité-Universitätsmedizin Berlin and the Martin Luther University Halle-Wittenberg. Written informed consent was obtained from all participants.

### Recruitment

Recruitment of the participants took place between 2014 and 2019 and was organized locally by the 18 study centers. Potential participants were randomly selected from local registration offices and invited in waves (à 500-1000 persons stratified for 10-year age groups: 10% of 20-29 and 30-39 years, respectively, 26,6% of 40-49, 50-59, and 60-69 years, respectively) to the study centers through a multistep invitation procedure. First, an invitation was sent by regular mail. This mail included an invitation letter, a flyer with a short description of the study objectives, a reference to the vote of the Data Protection Officer and the Ethics Committee, a letter of support from regional authorities, and a reply card. Potential participants could mark on the card if they were willing to participate as well as a phone number to be contacted to make an appointment. Participants could also call directly, write an e-mail or fax their reply card to the study center. If no contact could be established, after 14 and 28 days, a first and a second reminder letter were sent out, respectively. If there was no response after 2-3 months, in the study center Halle, a third reminder letter was sent out. In the study center Berlin, no third reminders were sent out. If the phone number of the potential participant was identified (e.g. via phone book or a phone number search service), they were directly contacted via phone by the study center following the invitation letter. After five unsuccessful attempts to reach the participant by phone, reminder letters were sent out, analogously to the potential participants without a known phone number. As an additional recruitment step, study centers could conduct face-to-face recruitment via home visits. However, as this type of recruitment is very time-consuming, this additional step was only carried out in two study centers of the NAKO: Berlin-Center and Halle (Saale), and just for a short time. A flowchart of the NAKO recruitment process is provided as a supplementary figure (Supplementary Fig. 1).

In the study center Halle, data from five recruitment waves were used. In each of the waves, new individuals were contacted. The recruitment waves 1-3 took place between July and October 2014, waves 4 and 5 took place between November 2014 and January 2015. During waves 4 and 5, home visits were performed instead of the third postal reminder. Home visits were usually conducted during the afternoon or during the weekend to increase the likelihood of reaching subjects who work during the day. The team of NAKO staff members consisted of two trained recruiters. Only one home visit per non-responder was attempted.

At the NAKO study center Berlin-Center, home visits were conducted from June 2016 to July 2017 among persons of assumed Turkish descent who had not answered the second reminder letter. We focused on persons with a Turkish background since they represent the largest migrant group (currently 2.8 million) in Germany and especially in Berlin since Germany invited so-called “guest workers” from predominantly Southern Europe and the Mediterranean region in the 1960s and 1970s [21]. The person’s Turkish background was not only based on their nationality (which is provided by the registration office) but additionally on his or her name since many persons with a migration background have German citizenship. One member of the study staff with a Turkish background and Turkish as a native language performed the home visits on one to 2 days per week for 1 year. The postal invitations sent before were only in the German language.

In both study centers, the procedure for home visits was almost identical. If nobody was met, an information card with contact details together with a study flyer (including a Turkish version in Berlin) was deposed in the mailbox. If a family member was encountered at home, this was documented, and the information card was handed out to this person. If the potential participant was at home, the study content was explained to rule out misunderstandings and fears in the hope to promote study participation. Since the study language was German, the participants should have a sufficient understanding of German to provide informed consent and responses in questionnaires. However, during the home visits, individual support was also offered and the possibility to have for example a family member with command of German to attend the participant in the study center. The aim of the home visits was to make directly an appointment for the examination in the study center or to receive at least the phone number of the potential participant to contact him/her later and arrange an appointment then. If a person declined study participation actively during the home visit, a non-responder questionnaire was provided, following the standard NAKO recruitment strategy.

### Variables and measures

Residents’ age (categorized into 5 age groups; 20-29, 30-39, 40-49, 50-59, 60-69), sex, and nationality were obtained from the city register beforehand and were therefore available for both participants and non-participants to report stratified response proportions. They were also assessed during baseline assessment via self-report.

Various socio-economic and health-related characteristics were assessed during the NAKO interview and included in the current analysis: marital status (dichotomized into living with a partner (being married or living together with a partner) or without a partner (separated, divorced, single, or widowed), education (dropped out of school, low (< 10 years), medium (10-12 years), and high (> 12 years)), employment status (categorized into employed (including fulltime, part-time/parental leave), retired, unemployed, and permanently disabled), and average income per household member. Health-related variables included lifetime prevalence of cardiovascular diseases (heart attack, angina pectoris, heart failure, cardiac arrhythmia, intermittent claudication, and stroke), chronic back pain, arthritis, osteoporosis, diabetes, cancer, and depression. These variables were recorded dichotomously (ever diagnosed by a physician (yes/no)).

For the sample recruited in Berlin-Center, additional variables were included: the place of birth (Germany or Turkey), native language (German yes or no), level of German language (dichotomized into good (including the answers very good and good), and weak (including the answers medium, bad, and very bad)), years since immigration (categorizes into 0-10 years, > 10 to 20 years, and > 20 years).

Health behaviors included smoking status and the body mass index (BMI). Smoking was assessed via a touchscreen-based self-administrated questionnaire and categorized into smokers, ex-smokers, and non-smokers. Body height and weight were measured at baseline using a calibrated integrated measurement station (SECA model 764, Seca®, Hamburg, Germany). Body mass index (BMI) was calculated from these measurements as weight over height squared in kg/m^2^, and categorized into normal weight (BMI 18.5 to < 25.0 kg/m^2^), overweight (BMI 25.0 to < 30.0 kg/m^2^), and obesity (BMI ≥30.0 kg/m2).

### Statistical analysis

Response proportion was calculated using the formula

$$ \frac{\mathrm{Participants}\ \mathrm{that}\ \mathrm{join}\mathrm{ed}\ \mathrm{the}\ \mathrm{study}}{\left(\mathrm{Participants}\ \mathrm{that}\ \mathrm{join}\mathrm{ed}\ \mathrm{the}\ \mathrm{study}+\mathrm{Subjects}\ \mathrm{that}\ \mathrm{refused}\ \mathrm{to}\ \mathrm{join}+\mathrm{Non}-\mathrm{responders}\right)}\ast 100\% $$

The response proportion includes as non-responders all eligible subjects in the denominator, even subjects that died or moved away before contact could be made, as we could not distinguish these possibilities. The participants’ demographic, socioeconomic, and medical characteristics were reported using common descriptive statistics. Continuous variables were reported as means with the standard deviation (SD), categorical variables were reported as absolute and relative frequencies. Relative frequencies were presented alongside their respective 95% confidence interval (CI). Sample characteristics were stratified according to recruitment efforts. All analyses were performed using the R statistical software version 3.2.3. Due to different sample characteristics and recruitment strategies, results from Halle (Saale) and Berlin-Center are reported separately.

To assess the effect of additional home visits on the composition of the final sample, we compared the educational levels of subjects with local respective census data of the so-called Mikrozensus 2011 [22]. For the census conducted in 2011 in Germany, the following data sources were used: a) register data from the Federal Employment Agency on all employees covered by social insurance for about 28.2 million persons, b) register data from public employers on civil servants, judges, and soldiers for around 1.97 million persons and c) data from the household survey (additional employment statistics) on a sample basis with about 7.9 million respondents including data from the survey at addresses with non-sensitive dormitories and shared accommodations with about 122,000 respondents. Because of stratified sampling in the NAKO (oversampling of persons > 40 years), we compare the proportion of persons with high school diplomas in the waves with and without home visits to census data in 10-year age strata.

## Results

### Home visits in the general population (NAKO study center Halle)

#### Recruitment process

**Fig. 1:**
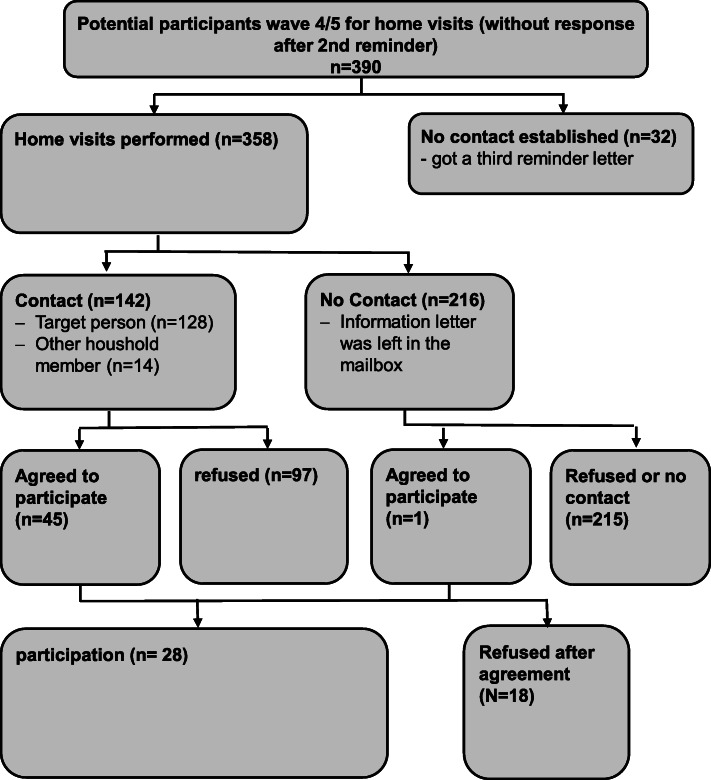
Recruitment flow-chart – NAKO study center Halle

In total, among 358 non-responders of the Halle study center from waves 4 and 5, a home visit was performed. In 142 visits (40%), the target person or a household member was encountered, in the remaining 60%, only information was left. In total, 28 (7.8%) new participants were recruited through this strategy, 27 of them resulted from the 142 cases when a person was encountered and only 1 of those 216 when nobody was encountered (Fig. 1).

#### Response

**Fig. 2:**
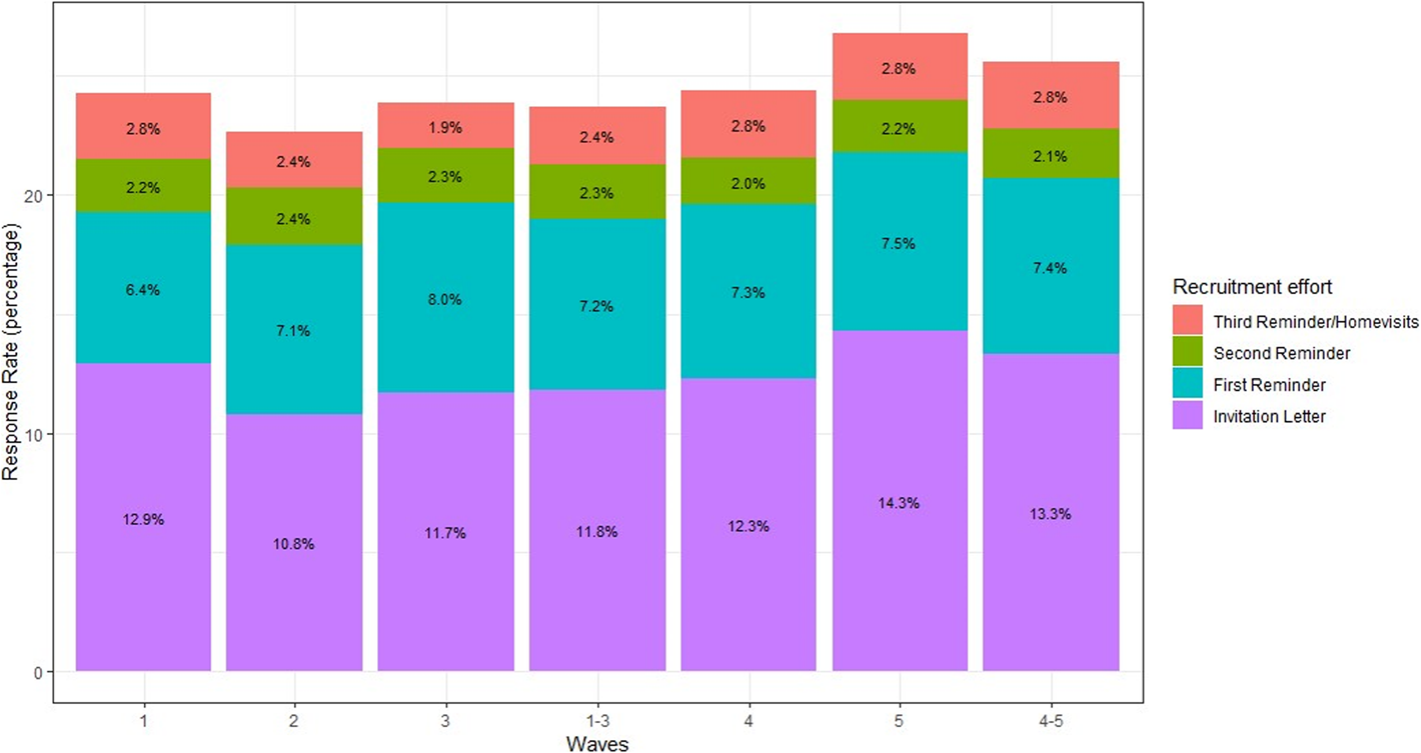
Response after different recruitment steps for waves 1-5 and summarized for waves 1-3 (third reminder letter) and waves 4-5 (home visits)

The response in waves 1-3 was increased from 21.3 to 23.6% by the third reminder letter. The overall response for waves 4-5 was increased from 22.8 to 25.6% by conducting the home visits (Fig. 2). The mean increase was thus 2.4 percentage points (95% CI: 1.7-3.3) for the third reminder letter and 2.8 percentage points (95% CI: 1.9-4.0) for home visits. Some gender and age differences in recruitment were observed: response was generally higher among women than among men in waves 1-5: 14.4% vs. 10.4% (first invitation), 7.8% vs. 6.7% (first reminder), 2.4% vs. 2.0% (second reminder), 1.2% vs. 1.7% (third reminder (waves 1-3)), and 1.1% vs. 1.1% (home visits (waves 4 and 5)), respectively. Concerning the different age groups, both the third reminder letter and home visits increased the response the most among the age group 20-29 (4.8 and 6.6% increase, respectively). The same age group had the highest response before the third reminder or home visit, indicating that at the overall low response additional recruitment efforts provide more participants in the same groups as already recruited.

#### Composition of the additionally recruited and final sample

Persons who were recruited during home visits were not different from those who were recruited in one of the precedent postal invitations (in the same wave) nor from those who participated after the third reminder letter (in previous waves). Minor differences were observed for some age groups, gender, marital status, smoking, and chronic diseases, but all of them were within the variation expected for the sample size (Supplementary Table 1).

Consistently, when comparing all participants recruited during waves 1-3 (invitation letter, 1st and 2nd reminder letter (standard recruitment), and additional 3rd reminder letter) with all participants recruited during waves 4-5 (standard recruitment and additional home visit), only small differences could be observed, which were within the expected variation for the sample size. For more details see Table 1.

**Table 1:** Characteristics of participants recruited in waves with postal mail only (wave 1-3) and waves including home visits (wave 4-5) in the Halle (Saale) NAKO study center

Recruitment steps	Postal mail only (waves 1-3) *N* = 359		Additional home visits (waves 4-5) *N* = 256	
Baseline variables	n	% (95%CI)	N	% (95%CI)
*Gender*				
Male	164	45.7 (40.6-50.9)	114	44.5 (38.6-50.6)
Female	195	54.3 (49.1-59.4)	142	55.5 (49.3-61.4)
*Age (years), mean ± SD*	*45.4 (14.8)*		*45.4 (14.3)*	
20 - 29	70	19.5 (15.7-23.9)	47	18.4 (14.1-23.6)
30 - 39	70	19.5 (15.7-23.9)	48	18.8 (14.4-24.0)
40 - 49	69	19.2 (15.5-23.6)	52	20.3 (15.8-25.7)
50 - 59	61	17.0 (13.5-21.2)	48	18.8 (14.4-24.0)
60 - 69	89	24.8 (20.6-29.5)	61	23.8 (19.0-29.4)
*Nationality*				
German	345	96.1 (93.6-97.7)	249	97.3 (94.5-98.7)
Non-German	14	3.9 (2.3-6.4)	7	2.7 (1.3-5.5)
*Marital Status*				
With partner	170	47.4 (42.2-52.5)	99	38.7 (32.9-44.8)
Without partner	189	52.6 (47.5-57.8)	157	61.3 (55.2-67.1)
*Employment Status*				
Fulltime/Part-time/Parental leave	219	71.8 (66.5-76.6)	170	75.9 (70.0-81.0)
Retired	55	18.0 (14.1-22.7)	33	14.7 (10.7-20.0)
Unemployed	28	9.2 (6.4-12.9)	18	8.0 (5.1-12.3)
Permanently disabled	3	0.9 (0.3-2.9)	3	1.3 (0.4-3.9)
*Average income per household member*				
< 500 euros	38	10.9 (8.1-14.6)	31	12.1 (8.7-16.7)
500 – 1000 euros	139	39.9 (34.9-45.2)	82	32.0 (26.6-38.0)
1000-2500 euros	157	45.1 (40.0-50.4)	124	48.4 (42.4-54.5)
2500-4000 euros	9	2.6 (1.4-4.8)	13	5.1 (3.0-8.5)
> 4000 euros	5	1.4 (0.6-3.3)	4	1.6 (0.6-3.9)
*Education*				
Dropped out of school	1	0.3 (0.0-1.6)	1	0.4 (0.0-2.2)
Low (< 10 years)	23	6.4 (4.3-9.4)	21	8.3 (5.5-12.3)
Middle (10-12 years)	135	37.6 (32.7-42.7)	105	41.3 (35.5-47.5)
High (> 12 years)	195	54.3 (49.1-59.4)	126	49.6 (43.5-55.7)
Other	5	1.4 (0.5-3.2)	3	1.2 (0.4-3.4)
*BMI, mean ± SD*		*26.6 (5.4)*		*26.8 (5.3)*
Normal weight (18.5 to < 25.0 kg/m^2^)	154	43.1 (38.1-48.3)	113	44.3 (38.3-50.5)
Overweight (25.0 to < 30.0 kg/m^2^)	127	35.6 (30.8-40.7)	82	32.2 (26.7-38.1)
Obesity (≥30.0 kg/m^2^)	76	21.3 (17.4-25.8)	60	23.5 (18.7-29.1)
*Smoking Status*				
Non-smoker	160	47.3 (42.1-52.7)	118	47.2 (41.1-53.4)
Ex-smoker	81	24.0 (19.7-28.8)	65	26.0 (21.0-31.8)
Current smoker	97	28.7 (24.1-33.7)	67	26.8 (21.7-32.6)
*Cardiovascular Diseases*				
Heart attack	9	2.5 (1.3-4.7)	3	1.3 (0.4-3.4)
Angina pectoris	14	3.9 (2.3-6.4)	6	2.3 (1.1-5.0)
Heart failure	16	4.4 (2.8-7.1)	8	3.1 (1.6-6.0)
Cardiac arrhythmia	49	13.6 (10.5-17.6)	20	7.8 (5.1-11.8)
Intermittent claudication	8	2.2 (1.1-4.3)	8	3.1 (1.6-6.0)
Stroke	4	1.1 (0.4-2.8)	3	1.3 (0.4-3.4)
*Other*				
Chronic back pain	76	21.2 (17.3-25.7)	52	20.3 (15.8-25.7)
Arthritis	51	14.2 (11.0-18.2)	48	18.8 (14.4-24.0)
Osteoporosis	11	3.2 (1.7-5.4)	10	3.9 (2.1-7.0)
Diabetes	27	7.5 (5.2-10.7)	16	6.3 (3.9-9.9)
Cancer	23	6.4 (4.3-9.4)	18	7.0 (4.5-10.8)
Depression	50	13.9 (10.7-17.9)	34	13.3 (9.7-18.0)

*CI* confidence interval, *SD* standard deviation, *BMI* body mass index

### Home visits among persons of Turkish descent (NAKO study center Berlin-center)

#### Recruitment process

**Fig. 3:**
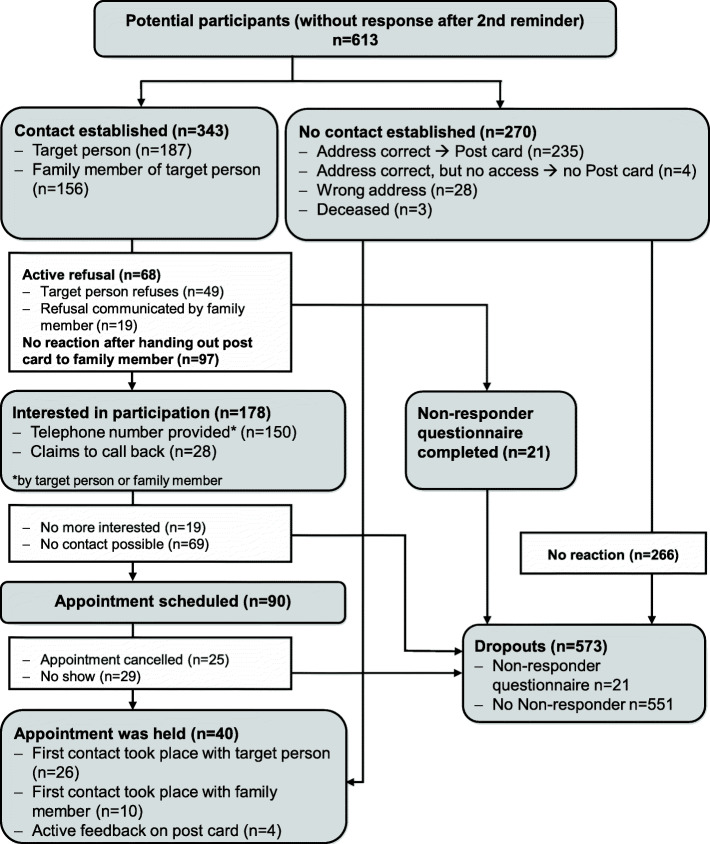
Recruitment flow-chart – NAKO study center Berlin-Center

Among all non-responders (after two reminders), 613 were classified as of Turkish descent and visited at home in the Berlin-Center region. 40 additional participants of Turkish descent were recruited through this strategy reflecting a response of 6.5% (Fig. 3). In comparison, 229 participants considered Turkish were recruited through postal invitation with up to two reminder letters in the corresponding recruitment waves. Since no onomastic characterization of all potential participants was performed before recruitment, no response increase due to home visits can be calculated for persons of Turkish descent.

#### Composition of the additionally recruited and final sample

In contrast to the home visits in the general population, in the population of Turkish descent, there were differences between those recruited via home visits and those recruited in preceding recruitment steps: those who were recruited via home visits were more often women, were more often born in Turkey, lived more often with a partner, and had more often a low educational level, less often reported German as their native language, and rated their German language skills as good. In addition, among the participants recruited via home visits, more were smokers, more had a low income, and fewer were employed. They were more often obese and reported more often having diabetes or depression. When comparing those recruited via home visits with those recruited in preceding postal invitations among the sample of Turkish descent, the effect of the home visits was small but remained visible. While the overall estimates for this subgroup were affected, the sample of those recruited via home visits was rather small and some of the observed differences might become more or less nuanced with a bigger sample.

For more details see Table 2.

**Table 2:** Characteristics of participants of Turkish descent recruited at various steps in the NAKO study center Berlin-Center

Recruitment steps	(Invitation and two reminder letters) *N* = 229		Additional home visits *N* = 40		Total *N* = 269	
Baseline variables	n	% (95%CI)	n	% (95%CI)	n	% (95%CI)
*Gender*						
Male	143	62.4 (56.0-68.5)	11	27.5 (16.1-42.8)	154	57.2 (51.3-63.0)
Female	86	37.6 (31.5-44.0)	29	72.5 (57.2-83.9)	115	42.8 (37.0-48.7)
*Age (years), mean ± SD*		*46.5 (11.7)*	*49.3 (11.0)*		*46.9 (11.6)*	
20 - 29	31	13.5 (9.7-18.6)	4	10.0 (4.0-23.1)	35	13.0 (9.5-17.6)
30 - 39	25	10.9 (7.5-15.6)	2	5.0 (1.4-16.5)	27	10.0 (7.0-14.2)
40 - 49	88	38.4 (32.4-44.9)	13	32.5 (20.1-48.0)	101	37.5 (32.0-43.5)
50 - 59	54	23.6 (18.5-29.5)	17	42.5 (28.5-57.8)	71	26.4 (21.5-32.0)
60 - 69	31	13.5 (9.7-18.6)	4	10.0 (4.0-23.1)	35	13.0 (9.5-17.6)
*Place of Birth*						
Born in Turkey	164	71.9 (65.8-77.4)	35	89.7 (76.4-95.9)	199	74.5 (69.0-79.4)
Born in Germany	64	28.1 (22.6-34.2)	4	10.3 (4.1-23.6)	68	25.5 (20.6-31.0)
*German as Native Language*						
No	170	74.2 (68.2-79.5)	33	84.6 (70.3-92.8)	203	75.7 (70.3-80.5)
Yes	59	25.8 (20.5-31.8)	6	15.4 (7.2-29.7)	65	24.3 (19.5-29.7)
*Level of German Language*						
Good (very good; good)	87	51.5 (44.0-58.9)	12	35.3 (21.5-52.1)	99	48.8 (42.0-55.6)
Weak (medium; bad; very bad)	82	48.5 (41.1-56.0)	22	64.7 (47.9-78.5)	104	51.2 (44.4-58.0)
*Years since immigration*						
0-10 years	6	3.9 (1.8-8.2)	2	6.3 (1.7-20.1)	8	4.3 (2.2-8.2)
> 10-20 years	17	11.0 (7.0-17.0)	4	11.8 (4.7-26.6)	21	11.1 (7.4-16.5)
> 20 years	131	85.1 (78.6- 89.8)	28	82.4 (66.5-91.7)	159	84.6 (78.7-89.0)
*Marital Status*						
With partner	130	56.8 (50.3-63.0)	31	77.5 (62.5-87.8)	161	60.1 (54.1-65.8)
Without partner	98	42.8 (36.6-49.3)	9	22.5 (12.3-37.5)	107	39.9 (34.2-45.9)
*Employment Status*						
Fulltime/Part-time/Parental leave	135	68.5 (61.7-74.6)	17	53.1 (36.4-69.1)	152	66.4 (60.0-72.2)
Retired	26	13.2 (9.2-18.6)	6	18.8 (8.9-35.3)	32	14.0 (10.1-19.1)
Unemployed	31	15.7 (11.3-21.5)	7	21.9 (11.0-38.8)	38	16.6 (12.3-22.0)
Permanently disabled	5	2.5 (1.1-5.8)	2	6.3 (1.7-20.1)	7	3.1 (1.5-6.2)
*Average income per household member*						
< 500 euros	56	28.3 (22.5-34.9)	14	38.9 (24.8-55.1)	70	29.9 (24.4-36.1)
500 – 1000 euros	83	41.9 (35.3-48.9)	18	50.0 (34.5-65.5)	101	43.2 (37.0-50.0)
1000-2500 euros	51	25.8 (20.2-32.3)	4	11.1 (4.4-25.3)	55	23.5 (18.5-29.3)
2500-4000 euros	3	1.5 (0.5-4.4)	0	0 (0-8.8)	3	1.3 (0.4-3.7)
> 4000 euros	5	2.5 (1.1-5.8)	0	0 (0-8.8)	5	2.1 (0.9-4.9)
*Education*						
Dropped out of school	21	9.2 (6.1-13.6)	7	17.5 (8.7-31.9)	28	10.4 (7.3-14.6)
Low (< 10 years)	66	28.8 (23.3-35.0)	25	62.5 (47.0-75.8)	91	33.8 (28.4-36.7)
Middle (10-12 years)	48	21.0 (16.2-26.7)	2	5.0 (1.4-16.5)	50	18.6 (14.4-23.7)
High (> 12 years)	85	37.1 (31.1-43.5)	4	10.0 (4.0-23.1)	89	33.1 (27.7-38.9)
missing	9	3.9 (2.1-7.3)	2	5.0 (1.4-16.5)	11	4.1 (2.3-7.2)
*BMI, mean ± SD*		*28.0 (4.7)*		*30.6 (5.5)*		*28.4 (4.9)*
Normal weight (18.5 to < 25.0 kg/m^2^)	55	25.6 (20.2-31.8)	7	17.5 (8.7-31.9)	62	24.3 (19.5-29.9)
Overweight (25.0 to < 30.0 kg/m^2^)	100	46.5 (40.0-53.2)	14	35.0 (22.1-50.5)	114	44.7 (38.7-50.8)
Obesity (≥30.0 kg/m^2^)	60	27.9 (22.3-34.3)	19	47.5 (32.9-62.5)	79	31.0 (25.6-36.9)
*Smoking Status*						
Non-smoker	72	40.7 (33.7-48.0)	7	35.0 (18.1-56.7)	79	40.1 (33.5-47.1)
Ex-smoker	41	23.2 (17.6-29.9)	4	20.0 (8.1-41.6)	45	22.8 (17.5-29.2)
Current smoker	64	36.2 (29.4-43.5)	9	45.0 (25.8-65.8)	73	37.1 (30.6-44.0)
*Cardiovascular Diseases*						
Heart attack	6	2.6 (1.2-5.6)	1	2.5 (0.4-12.9)	7	2.6 (1.3-5.3)
Angina pectoris	8	3.5 (1.8-6.7)	1	2.5 (0.4-12.9)	9	3.3 (1.8-6.2)
Heart failure	5	2.2 (0.9-5.0)	2	5.0 (1.4-16.5)	7	2.6 (1.3-5.3)
Cardiac arrhythmia	15	6.6 (4.0-10.5)	1	2.5 (0.4-12.9)	16	5.9 (3.7-9.4)
Intermittent claudication	3	1.3 (0.4-3.8)	0	0 (0-8.8)	3	1.1 (0.4-3.2)
Stroke	2	0.9 (0.2-3.1)	0	0 (0-8.8)	2	0.7 (0.2-2.7)
*Other*						
Chronic back pain	74	32.3 (26.6-38.6)	10	25.0 (14.2-40.2)	84	31.2 (26.0-37.0)
Arthritis	27	11.8 (8.2-16.6)	6	15.0 (7.1-29.1)	33	12.3 (8.9-16.7)
Osteoporosis	11	4.8 (2.7-8.4)	0	0 (0-8.8)	11	4.1 (2.3-7.2)
Diabetes	23	10.1 (6.8-14.6)	8	20.0 (10.5-34.8)	31	11.5 (8.2-15.9)
Cancer	4	1.7 (0.7-4.4)	2	5.0 (1.4-16.5)	6	2.2 (1.0-4.8)
Depression	54	23.6 (18.5-29.5)	14	35.0 (22.1-50.5)	68	25.3 (20.5-30.8)

*CI* confidence interval, *SD* standard deviation, *BMI* body mass index

### Comparison of the NAKO participants with the general population

The comparison with the census data showed that the participants recruited in the NAKO are much higher educated than the persons of the respective age group in the underlying populations (Tables 3 and 4). This is the case for both recruitment steps postal invitation with reminders and additional home visits, respectively. The complete tables including all education levels are presented in the supplement as Supplementary Tables 2 and 3. While the recruitment procedure included also a short assessment of non-respondents, the number of persons who decline participation but are willing to provide further information was very low (data not shown).

**Table 3:** Proportion of highly educated subjects (more than 12 years of education) among NAKO participants (Halle/Saale) and the respective census data [22]

Age groups	Waves (1-3) ***N =*** 359		Home visits waves (4-5) ***N =*** 256		Halle/Saale Census ***N =*** 177,110	
**20-29**	57	82.6 (72.0-89.8)	36	78.3 (64.4-87.7)	20,490	54.6 (54.1-55.1)
**30-39**	44	63.8 (52.0-74.1)	30	63.8 (49.5-76.0)	12,190	44.8 (44.2-45.4)
**40-49**	38	55.9 (44.1-67.1)	26	50.0 (36.9-63.1)	9410	29.9 (29.4-30.4)
**50-59**	19	31.1 (20.9-43.6)	18	38.3 (25.8-52.6)	8440	27.4 (26.9-27.9)
**Above 60**	37	42.5 (32.7-53.0)	16	26.2 (16.8-38.4)	16,910	33.8 (33.3-34.2)

**Table 4:** Proportion of highly educated subjects (more than 12 years of education) among NAKO participants of Turkish descent (Berlin-Center) and the respective census data [22]

Age groups	Invitation + reminders ***N =*** 229		Total (Invitation + Home visits) ***N =*** 269		Berlin Census ***N*** = 806,310	
**20-29**	20	64.5 (46.9-78.9)	22	64.7 (47.9-78.5)	72,740	40.6 (40.3-40.8)
**30-39**	16	64.0 (44.5-79.8)	16	59.3 (40.7-75.5)	74,670	36.1 (35.9-36.3)
**40-49**	29	34.5 (25.2-45.2)	31	32.0 (23.5-41.8)	47,850	28.1 (27.9-28.3)
**50-59**	12	24.0 (14.3-37.4)	12	17.9 (10.6-28.7)	31,840	27.7 (27.5-28.0)
**Above 60**	8	26.7 (14.2-44.4)	8	24.2 (12.8-41.0)	27,960	20.7 (20.5-20.9)

## Discussion

This study aimed to compare participants recruited via postal invitation and reminders with those recruited via additional home visits. Using data from two centers of NAKO, we demonstrated that there was little value in additional home visits as a part of the recruitment strategy among the general population. Response increased only marginally and those recruited via home visits were similar to those recruited either in earlier steps or by additional postal invitation. Overall, home visits did not improve the representativeness of the recruited sample. In contrast, there were some effects of home visits conducted among a population of Turkish descent. However, since the preceding recruitment steps did not use materials in the Turkish language, it is not clear how much of the effect can be attributed to home visits.

In the last decades, a decline in participation in epidemiological studies was observed; various possible explanations were proposed such as less social engagement in general, an increasing mistrust in research, and the fact that studies are more demanding than they used to be (e.g. taking several blood samples, long follow-up, multiple examinations) [1]. Also in our study, the overall response was low compared to earlier studies with about 26% in Halle and 17% in Berlin-Center (whole study sample, no specific data for persons of Turkish descent available) [7]. The effect of the additional home visits in a general population was only small and comparable to a third reminder letter, which is much less demanding in terms of efforts. Additional recruitments steps did not reduce the difference to the general population nor improve the basis for generating weighted estimates.

Home visits as an additional recruitment step have been investigated in several studies with heterogeneous results [16–18]. Effects regarding response increase seem to depend on sex, ethnicity, or if the population is generally healthy or a sample of patients. Non-participation of patients is often associated with disease severity resulting in higher participation of “healthier” patients and consequently in an underestimation of disease severity or an overestimation of effects of a certain treatment [23]. Among general population samples, evidence is less clear: a Cochrane review reported no increase of participation after performing home visits among a sample of women who were invited for a breast cancer screening [24]. Results of a recent meta-analysis investigating response in biobank studies showed, in contrast, that face-to-face contact as a recruitment measure could increase response notably [16].

Among persons with a migration background, home visits were described as useful by several authors. Ford et al. reported face-to-face recruitment at church as effective among African Americans, however, without reporting a comparison, since the face-to-face recruitment was conducted as an individual recruitment measure and not as an additional step after mailing or telephone contact [25]. In the pre-test phase of the NAKO, Reiss et al. showed that a 10% response could be reached among persons of Turkish descent using additional home visits and bilingual study staff and study material, however, the authors provide no information about response increase due to home visits or comparability with census data among this population group [26]. Similarly, Bonevski et al. propose bilingual study staff and material, adapted wording, and personal contact via face-to-face contact as recruitment strategies for vulnerable population groups or minorities [27], but they do not quantify the effect.

Home visits among the sample of the general population of Halle did not lead to a substantial increase in participation of other population groups than already recruited via postal invitations and reminders. This is in line with a recent study from the US conducted by the National Health Institute that did not find any differences regarding health status between early and late respondents in a population-based survey [28]. Several other studies, however, showed that early respondents were different from late respondents, who were rather young, had a lower educational level [29, 30], and showed a higher prevalence of unfavorable substance use such as smoking and drinking [30–34]. All above-mentioned studies, conducted between 2002 and 2013, had higher response proportions than the NAKO ranging from 35 to 52%, which could be one reason for larger differences between early and late respondents. An important result of the studies was that late respondents were different from early/intermediate respondents and active refusers as well, but could be considered as similar to non-respondents (if the information could be assessed using a non-responder questionnaire). Several studies showed that non-respondents had as well lower educational levels and more often unhealthy lifestyle behaviors than participants [33, 35–38]. We could not confirm these observations.

In contrast to the general sample, we found that in the subsample among persons of Turkish descent, home visits could reach persons who were different from those who had agreed to participate after one of the postal invitations. Home visits recruited persons were more often women, living together with their partner, were more often born in Turkey, had lower German language skills, lower-income, and lower education. This corresponds with results from Hernando et al. who showed that common reasons for non-participation are language barriers and cultural differences between study staff and participants (Hernando et al., 2018). Hughson et al. proposed home visits especially for recruitment of population groups with a “high likelihood of socioeconomic hardship” [39]. Unfavorable health behavior, as well as a higher prevalence of chronic diseases, are also factors that are generally found more frequently in non-participants as among our sample of Turkish migrants [38]. Those factors seem to be similar for baseline and follow-up participation. A recent study showed that re-participation at 6-year follow-up could be increased via home visits by 9.5% (from 40.6% after postal invitation and two reminders to 50.1%). Participants recruited via home visits were more often women, had more often their own migration experience, lower education, or were unemployed compared to the participants recruited via mailing and telephone contact [40]. However, when comparing our data with census data assessed in 2011 via the German “Mikrozensus 2011”, even if home visits among the Turkish sample led to the participation of less-educated persons, participants were still different from the underlying migrant population in Berlin. This is in line with other studies showing higher participation of persons with higher education and socio-economic status [2, 36, 38].

### Implications

Our results do not support a recommendation of home visits for the general population at current recruitment levels in Germany. Differences in participants recruited with and without home visits were so small that an effect on associations with exposures of interest would be very unlikely. While among persons with migration background, personal contact including native speaker seemed to promote the willingness of participation among those who were not recruited in preceding steps. However, it is not clear how much of this effect could be achieved by adding recruitment materials in the native language in postal invitations. It was not our aim to assess the representativeness of the NAKO sample in general, just to address the question of whether home visits have additional value for the final sample. Distortion of the distribution of some characteristics does not necessarily implicate bias, therefore, definitive judgment is not possible, but as far as we can infer from the studied characteristics, leaving out home visits did not introduce negative effects and the effort of home visits is not required.

### Strengths and limitations

One strength of our study is that it is embedded in a strongly standardized environment of the NAKO using recruitment via registration offices, implementing the same recruitment procedures in all study centers including training for study staff. This leads to good comparability between the two study centers Halle and Berlin-Center. Another strength is the inclusion of two different population samples in our analyses (general population and a subsample of Turkish descent) to compare the effect of home visits among those samples using the same methodology. A third strength is the wide spectrum of characteristics across which the differences could be assessed.

Some limitations have to be mentioned as well. First, our sample sizes of persons recruited via home visits were rather small, resulting in the low statistical power of our study. There is thus a probability that differences between groups or associations have not been detected. Second, no response increase caused by the home visits could be provided for the Turkish subsample, since the onomastic classification was not conducted for the standard recruitment responders. Furthermore, despite recommendations based on a NAKO pre-test, no bilingual study information was provided for persons with (a possible) migration background. We could therefore not separate the effects of home visits from the effects of providing information about the study in the native language. Also, the study materials were still in German, therefore the participation of persons without knowledge of German required additional support. This can be different when participation in a native language is offered. In addition, since the recruitment was performed in two urban settings without including more rural regions, the generalizability may be reduced.

## Conclusions

In the sample of the general population, home visits as an additional recruitment measure increased response only to a very limited degree and in a comparable dimension as a third reminder letter. Participants recruited via home visit did not differ from these recruited via regular mail. In a migrant sample, we show positive effects of home visits, but could not separate those from the effects of providing study materials with bilingual study information.

## Supplementary Information

**Additional file 1: Supplementary Figure 1.** Recruitment process of the German National Cohort (NAKO)

**Additional file 2: Supplementary Table 1.** Characteristics of participants recruited in waves 1-3 (invitation, 1st, and 2nd reminder vs. 3rd reminder) and participants recruited in waves 4-5 (invitation, 1st, and 2nd reminder vs. home visits)

**Additional file 3: Supplementary Table 2.** Comparison of educational levels between NAKO participants (Halle/Saale) and respective census data (all numbers are divided by within-group total)

**Additional file 4: Supplementary Table 3.** Comparison of educational levels between NAKO participants of Turkish descent (Berlin-Center) and respective census data (all numbers are divided by within-group total)

## Data Availability

The datasets used and/or analyzed during the current study are not yet publicly available due to the ongoing data management process but are available from the corresponding author on reasonable request.

## Abbreviations

BMI: Body mass index
CI: Confidence interval
NAKO: Nationale Kohorte
SD: Standard deviation
