# Supplementary material for: The effect of home visits as an additional recruitment step on the composition of the final sample: a cross-sectional analysis in two study centers of the German National Cohort (NAKO)
Source: BMC Med Res Methodol. 2021 Aug 23;21:176. doi: 10.1186/s12874-021-01357-z (PMC8383386; doi:10.1186/s12874-021-01357-z)
Supplement: Supplementary file 1 — Additional file 1: Supplementary Figure 1. Recruitment process of the German National Cohort (NAKO) [file 12874_2021_1357_MOESM1_ESM.pdf]

The effect of home visits as an additional recruitment step on the composition of the final sample: a cross-sectional analysis in two study centers of the German National Cohort (NAKO)

Lilian Krist*[1] & Ahmed Bedir**[2], Julia Fricke[1], Alexander Kluttig[3], Rafael Mikolajczyk[3]

[1] Institute of Social Medicine, Epidemiology and Health Economics, Charité-Universitätsmedizin, Berlin, Germany
[2] Department of Radiation Oncology, Health Services Research Group, University Hospital Halle (Saale), Halle (Saale), Germany.
[3] Institute of Medical Epidemiology, Biometry, and Informatics, Martin Luther University Halle-Wittenberg, Halle (Saale), Germany

*Corresponding author.
**Lilian Krist and Ahmed Bedir contributed equally to this manuscript.

Dr. Lilian Krist, lilian.krist@charite.de; https://orcid.org/0000-0002-6089-5163

Keywords: Response rate; response proportion; non-response bias; mixed mode design; recruitment strategy; home visits; Turkish, migrants.

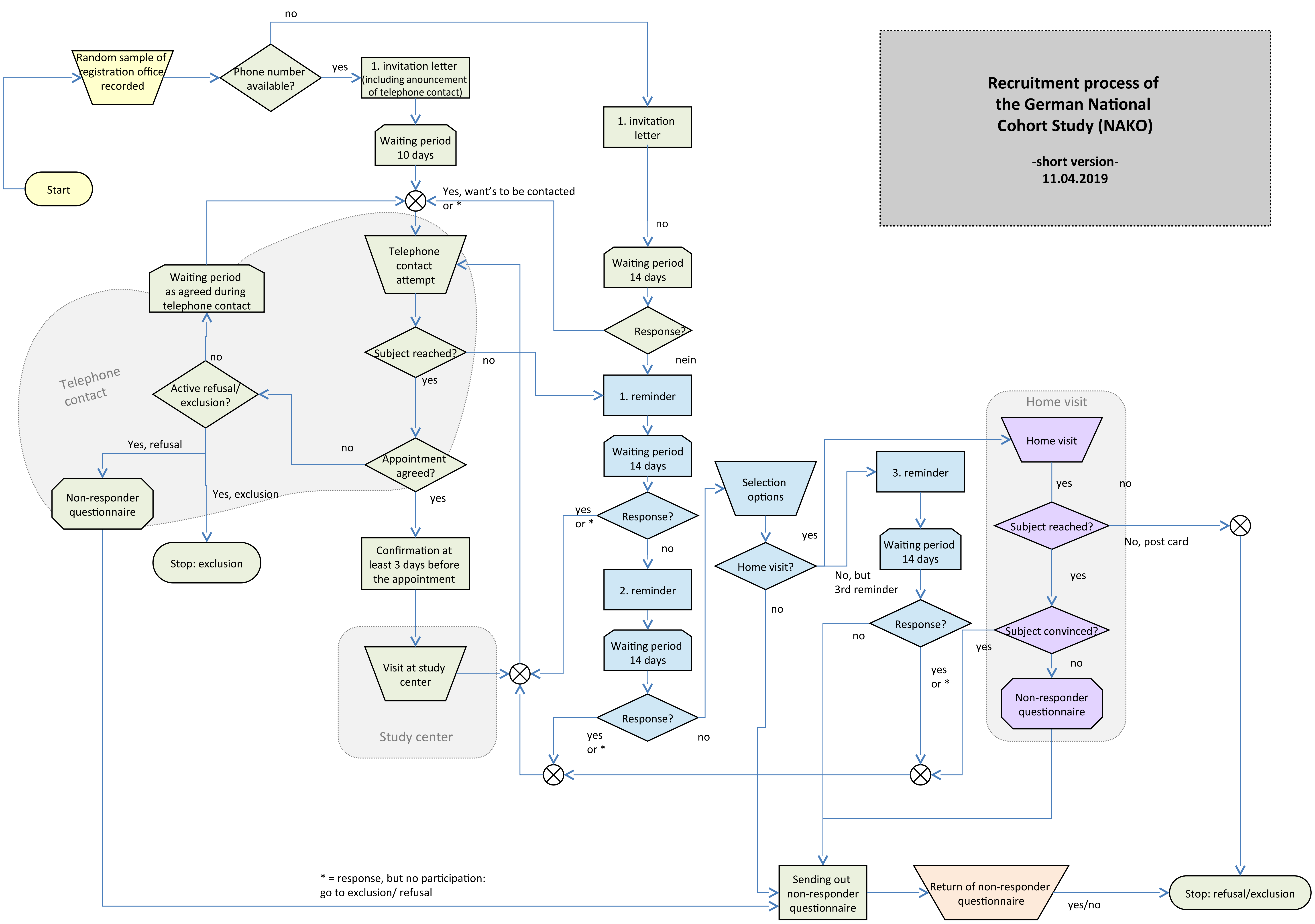
Recruitment process of the German National Cohort Study (NAKO)
-short version-
11.04.2019
Start
Random sample of registration office recorded
Phone number available?
yes
no
1. invitation letter (including anouncement of telephone contact)
Waiting period 10 days
Yes, want's to be contacted or *
Telephone contact attempt
Subject reached?
no
yes
Appointment agreed?
no
yes
Confirmation at least 3 days before the appointment
Visit at study center
Study center
Waiting period as agreed during telephone contact
Active refusal/ exclusion?
no
Yes, refusal
Yes, exclusion
Non-responder questionnaire
Stop: exclusion
Telephone contact
1. invitation letter
no
Waiting period 14 days
Response?
nein
1. reminder
Waiting period 14 days
Response?
yes or *
no
2. reminder
Waiting period 14 days
Response?
yes or *
no
Selection options
Home visit?
yes
no
No, but 3rd reminder
3. reminder
Waiting period 14 days
Response?
no
yes or *
Home visit
Home visit
yes
no
Subject reached?
No, post card
yes
Subject convinced?
yes
no
Non-responder questionnaire
Sending out non-responder questionnaire
Return of non-responder questionnaire
yes/no
Stop: refusal/exclusion
* = response, but no participation: go to exclusion/ refusal
