# Supplementary material for: The effect of home visits as an additional recruitment step on the composition of the final sample: a cross-sectional analysis in two study centers of the German National Cohort (NAKO)
Source: BMC Med Res Methodol. 2021 Aug 23;21:176. doi: 10.1186/s12874-021-01357-z (PMC8383386; doi:10.1186/s12874-021-01357-z)
Supplement: Supplementary file 2 — Additional file 2: Supplementary Table 1. Characteristics of participants recruited in waves 1-3 (invitation, 1st, and 2nd reminder vs. 3rd reminder) and participants recruited in waves 4-5 (invitation, 1st, and 2nd reminder vs. home visits) [file 12874_2021_1357_MOESM2_ESM.pdf]

The effect of home visits as an additional recruitment step on the composition of the final sample: a cross-sectional analysis in two study centers of the German National Cohort (NAKO)

Lilian Krist*[1] & Ahmed Bedir**[2], Julia Fricke[1], Alexander Kluttig[3], Rafael Mikolajczyk[3]

[1] Institute of Social Medicine, Epidemiology and Health Economics, Charité-Universitätsmedizin, Berlin, Germany
[2] Department of Radiation Oncology, Health Services Research Group, University Hospital Halle (Saale), Halle (Saale), Germany.
[3] Institute of Medical Epidemiology, Biometry, and Informatics, Martin Luther University Halle-Wittenberg, Halle (Saale), Germany

*Corresponding author.
**Lilian Krist and Ahmed Bedir contributed equally to this manuscript.

Dr. Lilian Krist, lilian.krist@charite.de; https://orcid.org/0000-0002-6089-5163

Keywords: Response rate; response proportion; non-response bias; mixed mode design; recruitment strategy; home visits; Turkish, migrants.

Supplementary Table 1. Characteristics of participants recruited in waves 1-3 (invitation, 1st, and 2nd reminder vs. 3rd reminder) and participants recruited in waves 4-5 (invitation, 1st, and 2nd reminder vs. home visits) in the NAKO study center Halle (Saale).

	Waves 1-3				Waves 4+5			
	(Invitation, 1st, and 2nd reminder) N= 323		(3rd reminder) N= 36		(Invitation, 1st, and 2nd reminder) N= 228		(additional home visits) N= 28	
	n	% (95%CI)	n	% (95%CI)	n	% (95%CI)	n	% (95%CI)
Gender								
Male	143	44.3 (38.9-49.7)	21	58.3 (42.2-72.9)	100	43.9 (37.6-50.3)	14	50.0 (32.6-67.3)
Female	180	55.7 (50.3-61.0)	15	41.7 (27.1-57.8)	128	56.1 (49.7-62.4)	14	50.0 (32.6-67.3)
Age (years), mean±SD		*45.9 (14.6)*		*40.7 (15.5)*		*45.9 (14.2)*		*41.9 (14.7)*
20 - 29	60	18.6 (14.7-23.2)	10	27.8 (15.8-44.0)	38	16.7 (12.4-22.0)	9	32.1 (17.9-50.7)
30 - 39	60	18.6 (14.7-23.2)	10	27.8 (15.8-44.0)	45	19.7 (15.1-25.4)	3	10.7 (3.7-27.2)
40 - 49	65	20.1 (16.1-24.8)	4	11.1 (4.4-25.3)	44	19.3 (14.7-24.9)	8	28.6 (15.3-47.1)
50 - 59	55	17.0 (13.3-21.5)	6	16.7 (7.9-31.9)	46	20.2 (15.5-25.9)	2	7.1 (2.0-22.6)
60 - 69	83	25.7 (21.2.-30.7)	6	16.7 (7.9-31.9)	55	24.1 (19.0-30.1)	6	21.4 (10.2-39.5)
Nationality								
German	312	96.7 (94.0-98.1)	33	91.7 (78.2-97.1)	223	97.8 (95.0-99.1)	26	92.9 (77.4-98.0)
Non-German	11	3.4 (1.9-6.0)	3	8.3 (2.9-21.8)	5	2.2 (0.9-5.0)	2	7.1 (2.0-22.6)
Marital Status								
With Partner	157	48.6 (43.2-54.0)	13	36.1 (22.5-52.4)	92	40.4 (34.2-46.8)	7	25.0 (12.7-43.3)
Without partner	166	51.4 (46.0-56.8)	23	63.9 (47.6-77.5)	136	59.6 (53.2-65.8)	21	75.0 (56.6-87.3)
Employment Status								
Fulltime/Part-time/Parental leave	202	62.5 (57.1-67.6)	17	63.0 (44.2-78.5)	155	76.7 (70.4-82.0)	15	68.2 (47.3-83.6)
Retired	52	16.1 (12.5-20.5)	3	11.1 (3.9-28.1)	29	14.4 (10.2-19.9)	4	18.2 (7.3-38.5)
Unemployed	22	6.8 (4.5-10.1)	6	22.2 (10.6-40.8)	15	7.4 (4.6-11.9)	3	13.6 (4.7-33.3)
Perm. Disabled	2	0.6 (0.2-2.2)	1	3.7 (0.7-18.3)	3	1.5 (0.5-4.3)	0	0 (0-14.9)
Average income per household member								
< 500 euros	30	9.3 (6.6-12.9)	8	22.2 (11.7-38.1)	28	12.3 (8.6-17.2)	3	11.5 (4.0-29.0)
500 – 1000 euros	122	37.8 (32.7-43.2)	17	47.2 (32.0-63.0)	71	31.1(25.5-37.4)	11	42.3 (25.5-61.1)
1000-2500 euros	147	45.5 (40.2-51.0)	10	27.8 (15.8-44.0)	114	50.0 (43.6-56.4)	10	38.5 (22.4-57.5)
2500-4000 euros	9	2.8 (1.5-5.2)	0	0 (0-9.6)	12	5.3 (3.0-9.0)	1	3.8 (0.7-19.0)
>4000 euros	4	1.2 (0.5-3.1)	1	2.8 (0.5-14.1)	3	1.3 (0.4-3.8)	1	3.8 (0.7-19.0)
Education								
Dropped out of school	1	0.3 (0.1-1.7)	0	0 (0-9.6)	1	0.4 (0.1-2.4)	0	0 (0-12.1)
Low	19	5.8 (3.8-9.0)	4	11.1 (4.4-25.3)	17	7.5 (4.7-11.6)	4	14.3 (5.7-31.5)
Middle	125	38.7 (33.6-44.1)	10	27.8 (15.8-44.0)	97	42.5 (36.3-49.0)	8	28.6 (15.3-47.1)
High	174	53.9 (48.3-59.4)	21	58.3 (42.2-72.9)	110	48.2 (41.8-54.7)	16	57.1 (39.1-73.5)

Other	4	1.2 (0.5-3.1)	1	2.8 (0.5-14.1)	3	1.3 (0.4-3.8)	0	0 (0-12.1)
BMI, mean±SD		*26.6 (5.5)*		*26.0 (4.9)*		*26.6 (5.1)*		*28.2 (6.8)*
Normal weight (18.5 to <25.0 kg/m^2)	137	42.7 (37.4-48.1)	17	47.2 (32.0-63.0)	101	44.5 (38.2-51.0)	12	42.9 (26.5-60.9)
Overweight (25.0 to <30.0 kg/m^2)	114	35.5 (30.5-40.9)	13	36.1 (22.5-52.4)	74	32.6 (26.8-38.9)	8	28.6 (15.3-47.1)
Obesity (≥30.0 kg/m^2)	70	21.8 (17.6-26.6)	6	16.7 (7.9-31.9)	52	22.9 (17.9-28.8)	8	28.6 (15.3-47.1)
Smoking Status								
Non-smoker	146	47.2 (41.8-52.8)	14	48.3 (31.4-65.6)	105	47.3 (40.8-53.9)	13	46.4 (29.5-64.2)
Ex-smoker	77	24.8 (20.4-30.0)	4	13.8 (5.5-30.6)	60	27.0 (21.6-33.2)	5	17.9 (7.9-35.6)
Current smoker	86	27.8 (23.1-33.1)	11	37.9 (22.7-56.0)	57	25.7 (20.4-31.8)	10	35.7 (20.7-54.2)
Cardiovascular Diseases								
Heart attack	7	2.2 (1.1-4.4)	2	5.6 (1.5-18.1)	2	0.9 (0.2-3.1)	1	3.6 (0.6-17.7)
Angina pectoris	13	4.0 (2.4-6.8)	1	2.8 (0.5-14.1)	5	2.2 (0.9-5.0)	1	3.6 (0.6-17.7)
Heart failure	14	4.3 (2.6-7.1)	2	5.6 (1.5-18.1)	5	2.2 (0.9-5.0)	3	10.7 (3.7-27.2)
Cardiac arrhythmia	44	13.6 (10.3-17.8)	5	13.9 (6.1-28.7)	16	7.0 (4.1-11.1)	4	28.6 (15.3-47.1)
Intermittent claudication	6	1.9 (0.9-4.0)	2	5.6 (1.5-18.1)	6	2.6 (1.2-5.6)	2	7.1 (2.0-22.6)
Stroke	2	0.6 (0.2-2.2)	2	5.6 (1.5-18.1)	2	0.9 (0.2-3.1)	1	3.6 (0.6-17.7)
Other								
Chronic Back Pain	70	21.7 (17.5-26.5)	6	16.7 (7.9-31.9)	44	19.3 (14.7-24.9)	8	28.6 (15.3-47.1)
Arthritis	47	14.6 (11.1-18.8)	4	11.1 (4.4-25.3)	42	18.4 (13.9-24.0)	6	21.4 (10.2-39.5)
Osteoporosis	11	3.4 (1.9-6.0)	0	0 (0-9.6)	10	4.4 (2.4-7.9)	0	0 (0-12.1)
Diabetes	23	7.1 (4.8-10.5)	4	11.1 (4.4-25.3)	10	4.4 (2.4-7.9)	6	21.4 (10.2-39.5)
Cancer	22	6.8 (4.5-10.1)	1	2.8 (0.5-14.1)	17	7.5 (4.7-11.6)	1	3.6 (0.6-17.7)
Depression	44	13.6 (10.3-17.8)	6	16.7 (7.9-31.9)	29	12.7 (9.0-17.7)	5	17.9 (7.9-35.6)

CI: confidence interval; SD=standard deviation; BMI=body mass index
