# Supplementary material for: The effect of home visits as an additional recruitment step on the composition of the final sample: a cross-sectional analysis in two study centers of the German National Cohort (NAKO)
Source: BMC Med Res Methodol. 2021 Aug 23;21:176. doi: 10.1186/s12874-021-01357-z (PMC8383386; doi:10.1186/s12874-021-01357-z)
Supplement: Supplementary file 3 — Additional file 3: Supplementary Table 2. Comparison of educational levels between NAKO participants (Halle/Saale) and respective census data (all numbers are divided by within-group total) [file 12874_2021_1357_MOESM3_ESM.pdf]

The effect of home visits as an additional recruitment step on the composition of the final sample: a cross-sectional analysis in two study centers of the German National Cohort (NAKO)

Lilian Krist*[1] & Ahmed Bedir**[2], Julia Fricke[1], Alexander Kluttig[3], Rafael Mikolajczyk[3]

[1] Institute of Social Medicine, Epidemiology and Health Economics, Charité-Universitätsmedizin, Berlin, Germany
[2] Department of Radiation Oncology, Health Services Research Group, University Hospital Halle (Saale), Halle (Saale), Germany.
[3] Institute of Medical Epidemiology, Biometry, and Informatics, Martin Luther University Halle-Wittenberg, Halle (Saale), Germany

*Corresponding author.
**Lilian Krist and Ahmed Bedir contributed equally to this manuscript.

Dr. Lilian Krist, lilian.krist@charite.de; https://orcid.org/0000-0002-6089-5163

Keywords: Response rate; response proportion; non-response bias; mixed mode design; recruitment strategy; home visits; Turkish, migrants.

Supplementary Table 2 Comparison of educational levels between NAKO participants (Halle/Saale) and respective census data (all numbers are divided by within group total)

Education per age group	Waves (1-3) N=359		Home visits waves (4-5) N=256		Halle/Saale Census N=177,110	
**20-29**						
Low	1	1.4 (0.3-7.8)	3	6.5 (2.2-17.5)	4,980	13.3 (12.9-13.6)
Middle	11	15.9 (9.1-26.3)	7	15.2 (7.6-28.2)	12,040	32.1 (31.6-32.3)
High	57	82.6 (72.0-89.8)	36	78.3 (64.4-87.7)	20,490	54.6 (54.1-55.1)
**30-39**						
Low	4	5.8 (2.2-14.0)	3	6.3 (2.2-17.2)	3,750	13.8 (13.4-14.2)
Middle	21	30.4 (20.8-42.1)	14	29.8 (18.7-44.0)	11,260	41.4 (40.8-42.0)
High	44	63.8 (52.0-74.1)	30	63.8 (49.5-76.0)	12,190	44.8 (44.2-45.4)
**40-49**						
Low	5	7.4 (3.2-16.1)	2	3.8 (1.1-13.0)	2,400	7.6 (7.3-7.9)
Middle	25	37.8 (26.3-48.6)	24	46.2 (33.3-59.5)	19,710	62.5 (62.0-63.0)
High	38	55.9 (44.1-67.1)	26	50.0 (36.9-63.1)	9,410	29.9 (29.4-30.4)
**50-59**						
Low	6	9.8 (4.6-19.8)	3	6.4 (2.2-17.2)	3,290	10.7 (10.3-11.0)
Middle	36	59.0 (46.5-70.5)	26	55.3 (41.2-68.6)	19,070	61.9 (61.4-62.5)
High	19	31.1 (20.9-43.6)	18	38.3 (25.8-52.6)	8,440	27.4 (26.9-27.9)
**Above 60**						
Low	8	9.2 (4.7-17.1)	11	18.0 (10.4-29.5)	18,220	36.4 (36.0-36.8)
Middle	42	48.3 (38.1-58.6)	34	55.7 (43.3-67.5)	14,950	29.9 (29.5-30.3)
High	37	42.5 (32.7-53.0)	16	26.2 (16.8-38.4)	16,910	33.8 (33.3-34.2)

Low: <10 years; middle: 10-12 years; high: >12 years.
