# Supplementary material for: The effect of home visits as an additional recruitment step on the composition of the final sample: a cross-sectional analysis in two study centers of the German National Cohort (NAKO)
Source: BMC Med Res Methodol. 2021 Aug 23;21:176. doi: 10.1186/s12874-021-01357-z (PMC8383386; doi:10.1186/s12874-021-01357-z)
Supplement: Supplementary file 4 — Additional file 4: Supplementary Table 3. Comparison of educational levels between NAKO participants of Turkish descent (Berlin-Center) and respective census data (all numbers are divided by within-group total) [file 12874_2021_1357_MOESM4_ESM.pdf]

The effect of home visits as an additional recruitment step on the composition of the final sample: a cross-sectional analysis in two study centers of the German National Cohort (NAKO)

Lilian Krist*[1] & Ahmed Bedir**[2], Julia Fricke[1], Alexander Kluttig[3], Rafael Mikolajczyk[3]

[1] Institute of Social Medicine, Epidemiology and Health Economics, Charité-Universitätsmedizin, Berlin, Germany
[2] Department of Radiation Oncology, Health Services Research Group, University Hospital Halle (Saale), Halle (Saale), Germany.
[3] Institute of Medical Epidemiology, Biometry, and Informatics, Martin Luther University Halle-Wittenberg, Halle (Saale), Germany

*Corresponding author.
**Lilian Krist and Ahmed Bedir contributed equally to this manuscript.

Dr. Lilian Krist, lilian.krist@charite.de; https://orcid.org/0000-0002-6089-5163

Keywords: Response rate; response proportion; non-response bias; mixed mode design; recruitment strategy; home visits; Turkish, migrants.

Supplementary Table 3 Comparison of educational levels between NAKO participants of Turkish descent (Berlin-Center) and respective census data (all numbers are divided by within group total).

**Education per age group**	**Invitation + reminders** N=229		**Total (Invitation + Home visits)** N=269		**Berlin Census** N=806,310	
**20-29**						
Low	4	12.9 (5.1-28.9)	5	14.7 (6.4-30.1)	43,720	24.4 (24.2-24.6)
Middle	7	22.6 (11.4-39.8)	7	20.6 (10.3-36.8)	62,850	35.1 (34.8-35.3)
High	20	64.5 (46.9-78.9)	22	64.7 (47.9-78.5)	72,740	40.6 (40.3-40.8)
**30-39**						
Low	5	20.0 (8.9-39.1)	6	22.2 (10.6-40.8)	63,770	30.8 (30.7-31.0)
Middle	4	16.0 (6.4-34.7)	5	18.5 (8.2-36.7)	68,270	33.0 (32.8-33.2)
High	16	64.0 (44.5-79.8)	16	59.3 (40.7-75.5)	74,670	36.1 (35.9-36.3)
**40-49**						
Low	34	40.5 (30.6-51.1)	44	45.4 (35.8-55.3)	69,670	40.9 (40.7-41.1)
Middle	21	25.0 (17.0-35.2)	22	22.7 (15.5-32.0)	52,770	31.0 (30.8-31.2)
High	29	34.5 (25.2-45.2)	31	32.0 (23.5-41.8)	47,850	28.1 (27.9-28.3)
**50-59**						
Low	29	58.0 (44.2-70.6)	46	68.7 (56.8-78.5)	48,900	42.6 (42.3-42.9)
Middle	9	18.0 (9.8-30.8)	9	13.4 (7.2-23.6)	34,040	29.7 (29.4-29.9)
High	12	24.0 (14.3-37.4)	12	17.9 (10.6-28.7)	31,840	27.7 (27.5-28.0)
**Above 60**						
Low	15	50.0 (33.2-66.8)	18	54.5 (38.0-70.2)	81,220	60.1 (59.8-60.3)
Middle	7	23.3 (11.8-40.9)	7	21.2 (10.7-37.8)	26,040	19.3 (19.0-19.5)
High	8	26.7 (14.2-44.4)	8	24.2 (12.8-41.0)	27,960	20.7 (20.5-20.9)

Low: <10 years; middle: 10-12 years; high: >12 years.
